# Correction to Enhancing Vibrational Light–Matter Coupling Strength beyond the Molecular Concentration Limit Using Plasmonic Arrays

**DOI:** 10.1021/acs.nanolett.1c01982

**Published:** 2021-06-01

**Authors:** Manuel Hertzog, Battulga Munkhbat, Denis Baranov, Timur Shegai, Karl Börjesson

The authors regret an error in the version of Figure 3 used in the published paper. The revised version of Figure 3 is shown here together with its figure caption.

**Figure 3:**
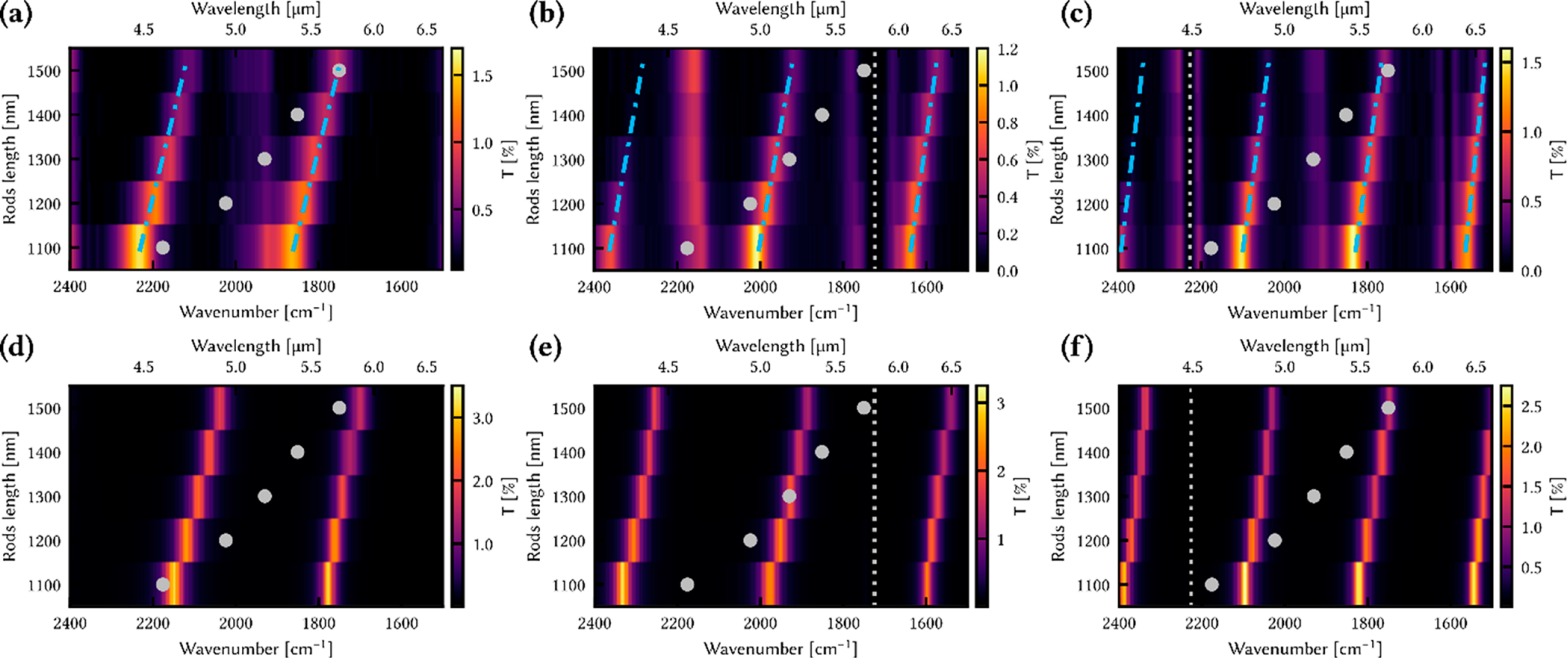
(a–c) Transmission maps of the gold rods inside the Fabry–Perot cavity, containing air, hexanal, or 4-butylbenzonitrile, respectively. All three were measured with a polarizer along the long axis of the rods. The blue dash-dot lines highlight the newly formed polaritonic states, and the residual vertical modes are an artifact due to nonideal polarization alignment. (d–f) Simulated spectra of the same systems (with the cavity thicknesses of 10.8, 8.5, and 10.4 μm, respectively). The gray dashed line indicates the absorption band of interest of the molecules, and the gray dots indicate the plasmon absorption maximum.

